# CircZDHHC20 represses the proliferation, migration and invasion in trophoblast cells by miR-144/GRHL2 axis

**DOI:** 10.1186/s12935-020-1097-2

**Published:** 2020-01-13

**Authors:** Bing Zhou, Xia Zhang, Ting Li, Rongping Xie, Jianbin Zhou, Yu Luo, Chunfen Yang

**Affiliations:** 1 Department of Obstetrics and Gynecology, The Second Affiliated Hospital, University of South China, Hengyang, Hunan China; 2 Department of Ultrasonography, The Second Affiliated Hospital, University of South China, Hengyang, Hunan China; 3 Department of Obstetrics and Gynecology, Guangdong Maternal and Child Health Hospital, Guangzhou, Guangdong China; 4 Department of Obstetrics and Gynecology, The First Affiliated Hospital, University of South China, No. 69 Chuanshan Road, Hengyang, 421001 Hunan China

**Keywords:** PE, circZDHHC20, miR-144, GRHL2, Trophoblast cells

## Abstract

**Background:**

Preeclampsia (PE) is a prevalent pregnancy disorder that has been one of the leading causes of maternal and perinatal mortality worldwide. Circular RNAs (circRNAs) have recently considered as important regulators in PE pathogenesis. In the current study, we aimed to explore the impact and mechanisms of circRNA zinc finger DHHC-type palmitoyltransferase 20 (circZDHHC20) in PE pathogenesis.

**Methods:**

RNase R assay and reverse transcription with Oligo(dT)_18_ primers were performed to confirm that circZDHHC20 was indeed circular transcript. The expression of circZDHHC20, grainyhead-like 2 (GRHL2) and miR-144 were assessed by quantitative real-time polymerase chain reaction (qRT-PCR). Subcellular localization assay was used to determine whether circZDHHC20 was predominantly present in the cytoplasm. The target correlations between miR-144 and circZDHHC20 or GRHL2 were confirmed using dual-luciferase reporter and RNA immunoprecipitation (RIP) assays. Cell proliferation, migration, and invasion were detected by 3-(4,5-dimethylthiazol-2-yl)-5-(3-carboxymethoxyphenyl)-2-(4-sulfophenyl)-2H-tetr-azolium (MTS), wound healing and transwell assays, respectively. Western blot was used for the quantification of GRHL2 protein level.

**Results:**

Our data indicated that circZDHHC20 was up-regulated and miR-144 was down-regulated in PE placenta. CircZDHHC20 sequestered miR-144 by acting as a miR-144 sponge. CircZDHHC20 overexpression repressed trophoblast cell proliferation, migration, and invasion, while its knockdown exerted opposite effects. Moreover, miR-144 mediated the regulation of circZDHHC20 on trophoblast cell behaviors. GRHL2 was directly targeted and inhibited by miR-144. MiR-144 exerted regulatory effects on trophoblast cell proliferation, migration and invasion by GRHL2. Furthermore, circZDHHC20 modulated GRHL2 expression through sponging miR-144.

**Conclusion:**

Our study suggested that a high level of circZDHHC20 inhibited the proliferation, migration, and invasion in trophoblast cells at least partially through sponging miR-144 and up-regulating GRHL2, providing a novel mechanism of PE pathogenesis.

## Highlights

1. CircZDHHC20 sequestered miR-144 by acting as a miR-144 sponge.
2. A high level of circZDHHC20 inhibited the proliferation, migration, and invasion in trophoblast cells.
3. GRHL2 was a direct target of miR-144, and circZDHHC20 modulated GRHL2 exoression by sponging miR-144.
4. CircZDHHC20 regulated trophoblast cell behaviors through miR-144/GRHL2 axis.

## Background

Preeclampsia (PE) is a prevalent pregnancy disorder that is often defined as new-onset hypertension and proteinuria during the second half of pregnancy [1, 2]. PE affects approximately 5% of pregnant women and has been one of the leading causes of maternal and perinatal mortality worldwide. Normal proliferation, migration, and invasion of trophoblast cells are fundamental in maintaining the function of human placenta [3]. Inadequate trophoblast cell behavior may be associated with PE pathogenesis [4]. However, the underlying mechanisms of PE pathogenesis are still largely unknown.

Circular RNAs (circRNAs), a novel class of non-coding RNA transcripts, have a special circular covalently bonded structure without a 5′ cap or a 3′ polyadenylated (pA) tail [5]. Owing to the inherent stability by their circular structure and exonuclease resistance, circRNAs have been considered as excellent disease biomarkers [6]. Emerging evidence has shown that circRNAs serve as important regulators in PE pathogenesis. For instance, Deng et al. found that has_circ_0011460 might be a novel potential therapeutic target for severe PE treatment using RNA sequencing [7]. Zhou et al. [8] reported that circRNA pregnancy-associated plasma protein A (circPAPPA) was down-regulated in PE placenta, and its deficiency hampered trophoblast cell proliferation and invasion . Shen et al. [9] manifested that circRNA trinucleotide repeat containing 18 (circTNRC18) was up-regulated in PE placenta, and a high circTNRC18 expression repressed the migration and epithelial-mesenchymal transition (EMT) of trophoblast cells in PE. Moreover, recent research demonstrated that circRNA zinc finger DHHC-type palmitoyltransferase 20 (circZDHHC20, hsa_circ_0006732) was up-regulated in maternal blood cells of PE patients, eliciting its potential involvement in PE pathogenesis [10]. Nevertheless, the impact and molecular mechanisms of circZDHHC20 in PE pathogenesis remain indistinct.

MicroRNAs (miRNAs) are endogenous small non-coding transcripts that have been widely accepted to play critical roles in the development of PE [11, 12]. Previous researches had described that miR-144 was low expressed in PE placenta and maternal plasma [13, 14], and miR-144 overexpression enhanced trophoblast cell proliferation, migration, and invasion [15]. In recent years, the competing endogenous RNA (ceRNA) hypothesis suggests that circRNAs could functionally interact with miRNAs, illuminating the importance of such interaction in PE pathogenesis [16, 17]. Intriguingly, two putative binding sites of miR-144 and circZDHHC20 (hsa_circ_0029698) or grainyhead-like 2 (GRHL2) were identified by the computational methods, eliciting the possibly regulatory network of the circZDHHC20/miR-144/GRHL2 axis. In the current study, we aimed to explore the impact of circZDHHC20 in PE pathogenesis and underlying mechanisms governing it.

## Materials and methods

### Clinical specimens and cell culture

Specimen collection and analysis processes were approved by the Ethics Committee of The Second Affiliated Hospital, University of South China. For this study group, placental tissues were collected from 15 healthy volunteers following a normal course of pregnancy and 26 PE patients, who underwent delivery at the Department of Obstetrics and Gynecology of The Second Affiliated Hospital, University of South China. Women with inflammatory disease, cardiovascular disease, chronic renal disease, diabetes mellitus, or recent infection were excluded from this study. All participators were asked to sign and confirm the written informed consent.

Human extravillous trophoblast HTR-8/SVneo cells were obtained from the American Type Culture Collection (ATCC, Manassas, VA, USA) and grown at 37 °C in an atmosphere of 5% CO_2_/95% air in Dulbecco’s Modified Eagle Medium/Nutrient Mixture F-12 (DMEM/F-12, Gibco, Tokyo, Japan) containing 10% (v/v) fetal calf serum (FCS, Gibco), 100 U/mL penicillin, and 100 µg/mL streptomycin.

### RNA extraction and RNase R digestion

Total RNA was obtained using TRIzol reagent (Ambion, Thermo Fisher Scientific, Paisley, UK) from placental tissues and HTR-8/SVneo cells. In RNase R assay, RNA extracts (100 µg) were incubated with RNase R (3 U/µg, Epicenter Technologies, Madison, WI, USA) at 37 °C for 20 min, followed by the purification using the RNeasy MinElute Cleanup Kit (Qiagen, Tokyo, Japan), referring to the producer’s guidance.

### Reverse transcription and quantitative real-time polymerase chain reaction

Random or Oligo(dT)_18_ primers were used for reverse transcription polymerase chain reaction (PCR) with the RNA to cDNA EcoDry Premix Kit (Clontech, Otsu, Japan). The expression of circZDHHC20, linear ZDHHC20 mRNA, and GRHL2 mRNA were analyzed by quantitative real-time PCR (qRT-PCR) using SYBR Green PCR Kit (Qiagen) on a Rotor-Gene Q real-time PCR System (Qiagen), with the housekeeping gene 18S rRNA as the internal control. The quantification of miR-144 was carried out using the miScript II Reverse Transcription Kit (Qiagen) and miScript SYBR Green PCR Kit (Qiagen), and U6 level was used as the loading control. PCR primers used for PCR amplification were listed: circZDHHC20 sense: 5′-CCTACATTGACATGTACACAGAACA-3′, circZDHHC20 antisense: 5′-TTCCACTGATCATTTTCTTGC-3′; linear ZDHHC20 mRNA sense: 5′-CGGCAACCCCTTTATGACTA-3′, linear ZDHHC20 mRNA antisense: 5′-CCACTCACTGGAAGCAATCA-3′; miR-144 sense: 5′-ATCCAGTGCGTGTCGTG-3′, miR-144 antisense: 5′-TGCTTATACAGTATAGATG-3′; GRHL2 mRNA sense: 5′-GGGCATAGGACTCCAGAGTAGGAA-3′, GRHL2 mRNA antisense: 5′-TAGGGCAGGACTGGCAAACA-3′; 18S rRNA sense: 5′-GGTCCGTGTTTCAAGACGG-3′, 18S rRNA antisense: 5′-GCATATCAATAAGCGGAGGAA-3′; U6 sense: 5′-GCTTCGGCAGCACATATACTAA-3′, U6 antisense: 5′-AACGCTTCACGAATTTGCGT-3′.

### Subcellular fractionation

Cytoplasmic and Nuclear RNA Purification Kit (Norgen Biotek, Thorold, ON, Canada) was used to isolate and purify the RNA from nuclear and cytoplasm fractions. After that, the expression levels of circZDHHC20, 18S rRNA and U6 in nuclear and cytoplasm fractions of HTR-8/SVneo cells were assessed by qRT-PCR assay.

### Cell transfection

The sequences of circZDHHC20 and GRHL2 were cloned into the pcDNA3.1 vector (Promega, Toyko, Japan) to construct the corresponding overexpression plasmid, respectively, and nontarget pcDNA3.1 vector was used as the negative control. The modified miR-144 mimic (5′-UCAUGUAGUAGAUAUGACAU-3′), mimic negative control (miR-con, 5′-UUCUCCGAACGUGUCACGUUU-3′), inhibitor of miR-144 (anti-miR-144, 5′-AUGUCAUAUCUACUACAUGA-3′), inhibitor negative control (anti-miR-con, 5′-CAGUACUUUUGUGUAGUACAA-3′), siRNA against circZDHHC20 (si-circZDHHC20, 5′-AAAAUACCAGUCUAUAAUGUU-3′) and GRHL2 (si-GRHL2, 5′-AGGUAAUUCUGCUUUUCCGUC-3′), and a scrambled negative control sequence (si-con, 5′-UUCUCCGAACGUGUCACGU-3′) were obtained from GenePharma (Shanghai, China). The Lipofectamine^TM^ 3000 Transfection Reagent was purchased from Thermo Fisher Scientific and used for each transfection following the protocols of manufacturers.

### Bioinformatics

Analysis for the directly interacted miRNAs of circZDHHC20 was carried out using the website tool Circular RNA Interactome (CircInteractome) (https://circinteractome.nia.nih.gov/miRNA_Target_Sites/mirna_target_sites.html). The potential molecular targets of miR-144 were identified using the DIANA-microT-CDS server (http://diana.imis.athena-innovation.gr/DianaTools/index.php?r=microT_CDS/index).

### Dual-luciferase reporter assay

The specific sequences of circZDHHC20 and GRHL2 3′-untranslated region (3′-UTR) harboring the complementary site of miR-144 were cloned into the pmirGLO vector (Promega) to generate the corresponding wild-type luciferase reporter constructs (circZDHHC20-WT and GRHL2-WT), respectively. To generate circZDHHC20 and GRHL2 mutant-type reporter constructs, the Q5 Site-directed Mutagenesis Kit (New England Biolabs, Ipswich, MA, USA) was used to mutate the target site. After that, the constructs were introduced into HTR-8/SVneo cells, respectively, together with miR-con mimic, miR-144 mimic, anti-miR-con, or anti-miR-144. 24 h later, the cells were solubilized and luciferase assays were implemented using the PicaGene Dual SeaPansy Luminescence Kit (Toyo Inki, Tokyo, Japan) and an ARVO X4 plate reader (PerkinElmer, Yokohama, Japan).

### RNA immunoprecipitation (RIP) assay

HTR-8/SVneo cells were homogenized in RIPA lysis buffer (Elabscience, Wuhan, China) supplemented with protease inhibitor cocktails (Thermo Fisher Scientific). Cell lysates were incubated with magnetic bead-coupled anti-Argonaute2 antibody (anti-Ago2, Abcam, Cambridge, UK) or a negative control IgG antibody (Abcam), following the instructions of the Magna RNA Immunoprecipitation Kit (Millipore, Zug, Switzerland). Total RNA was isolated from the beads and subjected to qRT-PCR for the enrichment of circZDHHC20, miR-144, and GRHL2.

### Cell proliferation assay

HTR-8/SVneo cells were seeded into 96-well plates and transfected with the indicated oligonucleotides or/and plasmids for 0, 24, 48 and 72 h. At each time point, the analysis of cell proliferation was performed by adding 20 µl of 3-(4,5-dimethylthiazol-2-yl)-5-(3-carboxymethoxyphenyl)-2-(4-sulfophenyl)-2H-tetr-azolium (MTS) reagent provided by the CellTiter 96^®^ AQ ueous One Solution Cell Proliferation Assay Kit (Promega). The number of viable cells was proportional to the absorbance at 490 nm which was read by the FLUOstar Omega microplate reader (BMG Labtech, Ortenberg, Germany).

### Wound healing assay

Migration ability of HTR-8/SVneo cells after transfection was assessed using a wound healing assay. In brief, cells (1.0 × 10^6^) were placed in a 6-well plate and subjected to various transfections. Then, a scratch was made in the cell monolayer using a sterile 200-µl pipette tip. The images of 0 and 48 h were photographed by an inverted microscope (Eclipse TE200, Nikon, Toyko, Japan) at 100 × magnification. Lastly, the Image-ProPlus digitizing system (Media Cybernetics, Buckinghamshire, UK) was used to detect the distance of cell migration.

### Transwell invasion assay

Inserts containing 8-µm pores in 24-transwell plates (BD Biosciences, North Ryde, NSW, Australia) were coated with Matrigel (BD Biosciences). Next, transfected cells (5.0 × 10^4^) in serum-free media were seeded into the Matrigel-precoated inserts, and 500 µl grown medium plus 10% FCS was added into the lower chamber. The cells that invaded through the pores of inserts were fixed with methanol and stained with 1% crystal violet. A 200 × Nikon magnification microscope was used to photograph the invaded cells in three random fields.

### Western blot for GRHL2 level

Transfected HTR-8/SVneo cells were lysed in RIPA lysis buffer as described above, and total protein was quantified by using a BCA Protein Assay Kit (Thermo Fisher Scientific). Protein extracts (~ 100 µg) were electrophoresed on an 8% SDS polyacrylamide gel, transferred to the nitrocellulose membranes (Amersham, Buckinghamshire, UK), and blocked in 3% (w/v) non-fat dry milk in PBS. For primary antibodies, we used rabbit anti-GRHL2 antibody (1:1000, Abcam) or rabbit anti-β-actin antibody (1:5000, Abcam). Horseradish peroxidase (HRP)-conjugated goat anti-rabbit IgG (1:10,000, Abcam) was used as the secondary antibody. Immunoreactive bands were determined using the Enhanced Chemiluminescence Detection Kit (Millipore).

### Statistical analysis

Significance between two data was analyzed by a two-tail Student’s *t* test. Multiple group experiments were compared using one-way analysis of variance (ANOVA), followed by Bonferroni’s multiple comparison test. Correlations between circZDHHC20, miR-144 and GRHL2 expression in placental tissues from PE patients using the Spearman test. All results were reported as mean ± standard deviation (SD). Statistical significance is denoted by *P < 0.05.

## Results

### CircZDHHC20 was up-regulated and miR-144 was down-regulated in placental tissues from PE patients

**Fig. 1:**
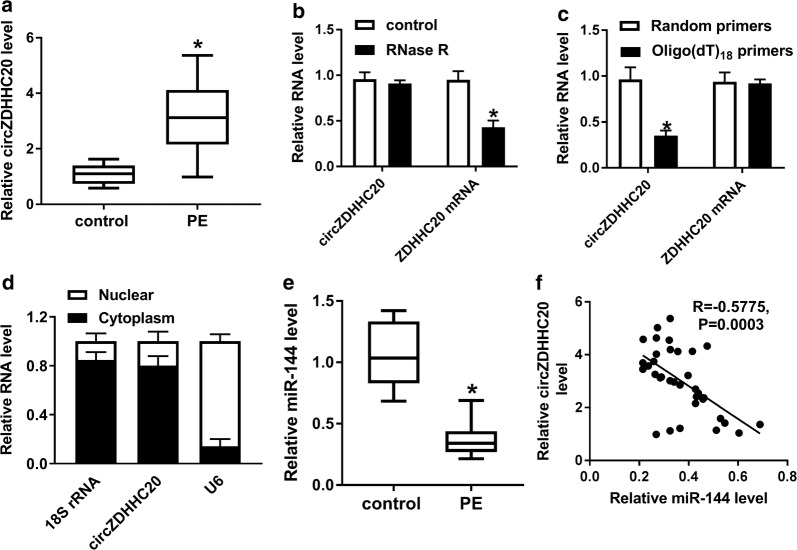
CircZDHHC20 was up-regulated and miR-144 was down-regulated in PE placental tissues. **a** qRT-PCR for circZDHHC20 expression in placental tissues from 26 PE patients and 15 healthy volunteers. **b** qRT-PCR for the levels of circZDHHC20 and linear ZDHHC20 mRNA after RNase R digestion. **c** The expression of circZDHHC20 and linear ZDHHC20 mRNA by qRT-PCR in reverse transcription using Random and Oligo(dT)_18_ primers. **d** CircZDHHC20 level by qRT-PCR in the nuclear and cytoplasm fractions of HTR-8/SVneo cells. 18S rRNA and U6 were used as internal controls. **e** The level of miR-144 in placental tissues from 26 PE patients and 15 healthy volunteers. **f** The correlation between circZDHHC20 level and miR-144 expression in placental tissues from 26 PE patients using the Spearman test. *P < 0.05

Firstly, we determined the expression pattern of circZDHHC20 in placental tissues from PE patients and healthy volunteers. As shown by qRT-PCR, circZDHHC20 level was higher in PE group than that in control group (Fig. 1a). To confirm that circZDHHC20 was indeed circular transcript, RNase R assay was performed. These results revealed that linear transcript was significantly digested by RNase R and circZDHHC20 was resistant to RNase R digestion (Fig. 1b). Because circRNAs were depleted in the 3′ pA tail, we used Random and Oligo(dT)_18_ primers in reverse transcription experiments, respectively. As expected, circZDHHC20 level was lower compared with linear transcript (Fig. 1c). Additionally, the data of subcellular localization assay showed that circZDHHC20 was highly enriched in the cytoplasm fraction in HTR-8/SVneo cells (Fig. 1d). qRT-PCR results also demonstrated that miR-144 expression was prominently reduced in placental tissues from PE patients compared to those of healthy volunteers (Fig. 1e). Besides, an inverse correlation between circZDHHC20 level and miR-144 expression was found in PE placental tissues (Fig. 1f).

### CircZDHHC20 sequestered miR-144 by acting as a miR-144 sponge

**Fig. 2:**
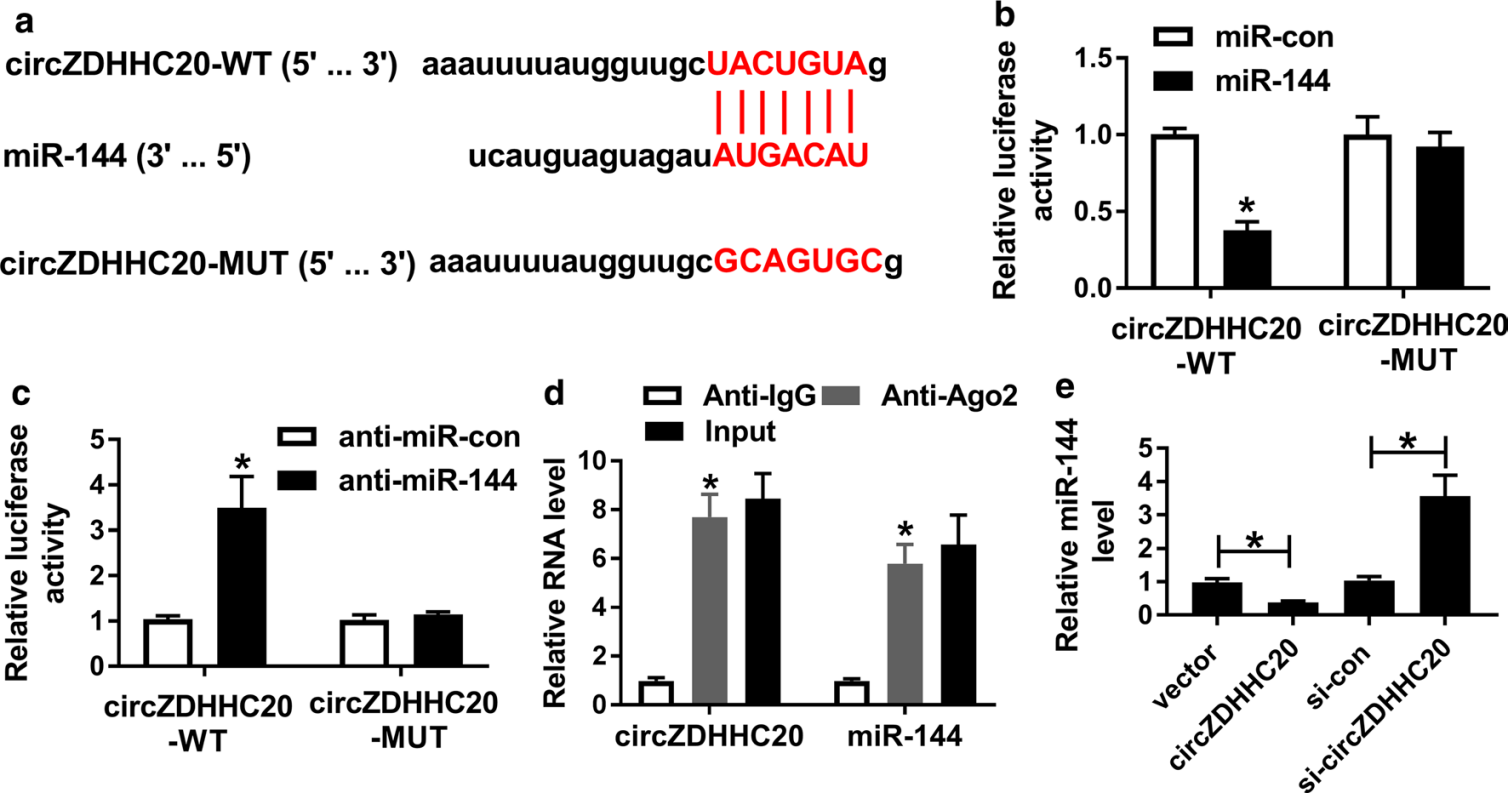
CircZDHHC20 acted as a molecular sponge of miR-144. **a** Schematic of circZDHHC20 illustrating position of the miR-144-binding site and mutated the miR-144-binding site. **b**, **c** Relative luciferase activity in HTR-8/SVneo cells cotransfected with circZDHHC20-WT or circZDHHC20-MUT and miR-con mimic, miR-144 mimic, anti-miR-con or anti-miR-144. **d** The enrichment of circZDHHC20 and miR-144 in the RISC of HTR-8/SVneo cells using anti-Ago2 or IgG antibody, with Input content as positive control. **e** The expression of miR-144 by qRT-PCR in HTR-8/SVneo cells transfected with negative control plasmid (vector), circZDHHC20 overexpression plasmid (circZDHHC20), si-con or si-circZDHHC20. *P < 0.05

CircRNAs prominently located in the cytoplasm are considered to regulate the abundance of available miRNAs through sponging miRNAs [16, 18]. Herein, we further observed whether circZDHHC20 could act as miRNAs sponges. Using CircInteractome computational method, a putative complementary site for miR-144 was found in circZDHHC20 (Fig. 2a). To verify whether circZDHHC20 served as a molecular sponge of miR-144, dual-luciferase reporter and RIP assays were implemented in HTR-8/SVneo cells. In dual-luciferase report assay, we cloned the partial sequences of circZDHHC20 harboring the miR-144-binding site into a luciferase plasmid and mutated the miR-144-binding site. In comparison to a corresponding negative control, the luciferase activity of wild-type reporter was significantly reduced by miR-144 overexpression, while it was highly elevated after miR-144 deficiency (Fig. 2b, c). However, when the target site was mutated, little change in luciferase was observed with either miR-144 overexpression or knockdown (Fig. 2b, c). In RIP experiments, anti-Ago2 antibody was used to bind to the RNA-induced silencing complex (RISC) [19]. These results revealed that circZDHHC20 and miR-144 were simultaneously enriched by anti-Ago2 antibody compared with IgG antibody (Fig. 2d), eliciting a possibility that circZDHHC20 could bind to miR-144 in the RISC. Moreover, the data of qRT-PCR showed that in contrast to their counterparts, miR-144 expression was prominently decreased by circZDHHC20 overexpression and increased when circZDHHC20 depletion in HTR-8/SVneo cells (Fig. 2e), suggesting that the miR-144-binding site was functional.

### MiR-144 mediated the regulation of circZDHHC20 on proliferation, migration and invasion in trophoblast cells

**Fig. 3:**
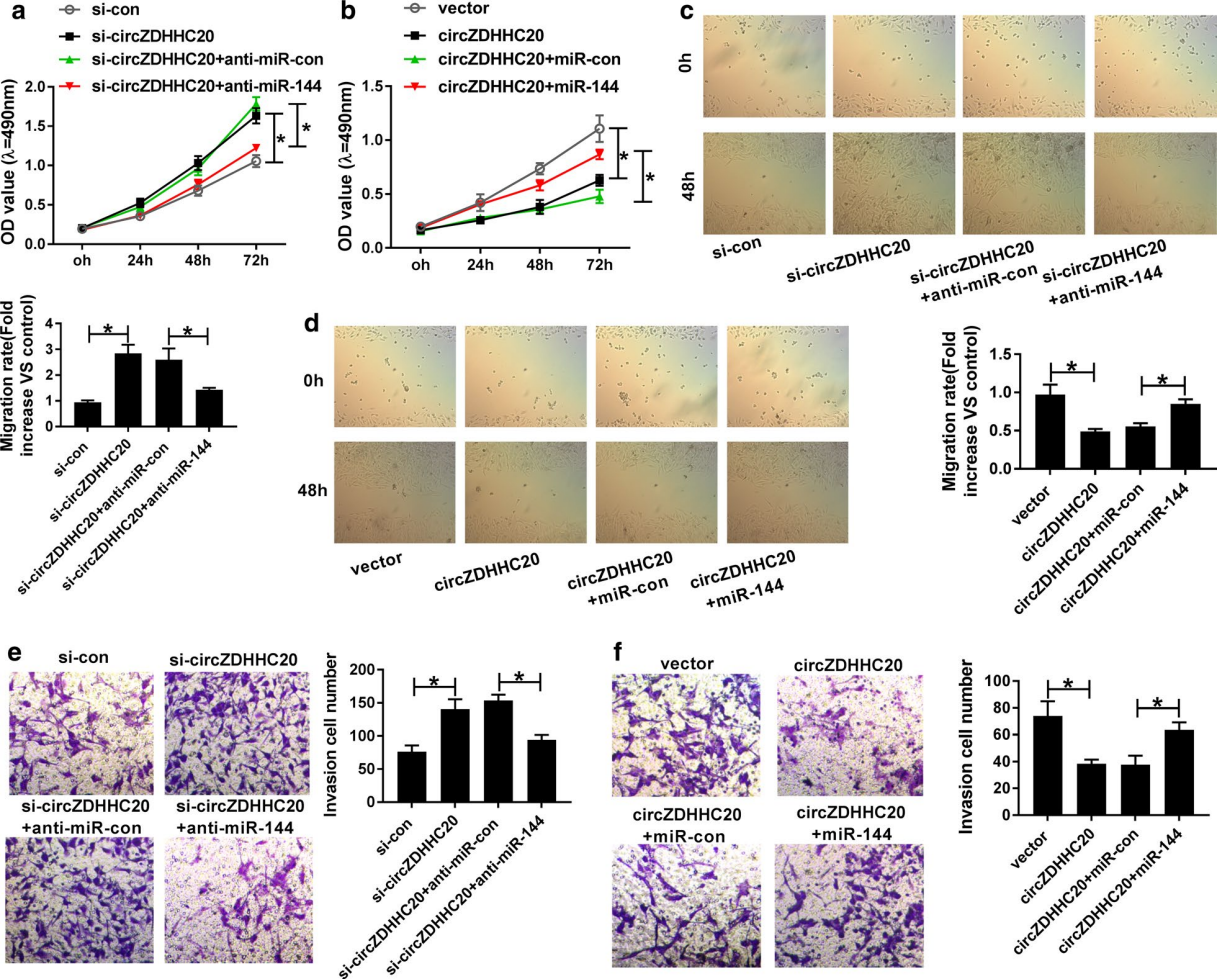
MiR-144 was involved in the regulation of circZDHHC20 on trophoblast cell proliferation, migration, and invasion. MTS assay for cell proliferation (**a**, **b**), wound healing assay for cell migration (**c**, **d**), transwell assay for cell invasion (**e**, **f**) in HTR-8/SVneo cells transfected with si-con, si-circZDHHC20, si-circZDHHC20 + anti-miR-con, si-circZDHHC20 + anti-miR-144, negative control plasmid (vector), circZDHHC20 overexpression plasmid (circZDHHC20), circZDHHC20 + miR-con mimic, or circZDHHC20 + miR-144 mimic. *P < 0.05

To explore the functional role of circZDHHC20 in PE pathogenesis, we manipulated its expression by siRNA targeting circZDHHC20 (si-circZDHHC20) and circZDHHC20 overexpression plasmid. MTS assays revealed that compared with a corresponding control, cell proliferation was significantly enhanced by circZDHHC20 knockdown (Fig. 3a) and weakened when circZDHHC20 up-regulation (Fig. 3b). Moreover, the results of wound healing and transwell assays showed that circZDHHC20 silencing led to the significant promotion in cell migration and invasion, while its overexpression exhibited opposite effects (Fig. 3c–f).

Then, we determined whether circZDHHC20 regulated trophoblast cell proliferation, migration, and invasion by miR-144. As expected, in contrast to their counterparts, the regulatory effects of circZDHHC20 knockdown and overexpression on cell proliferation, migration and invasion were significantly abolished by the introduction of anti-miR-144 or miR-144 mimic in HTR-8/SVneo cells (Fig. 3a–f).

### GRHL2 was directly targeted and inhibited by miR-144

**Fig. 4:**
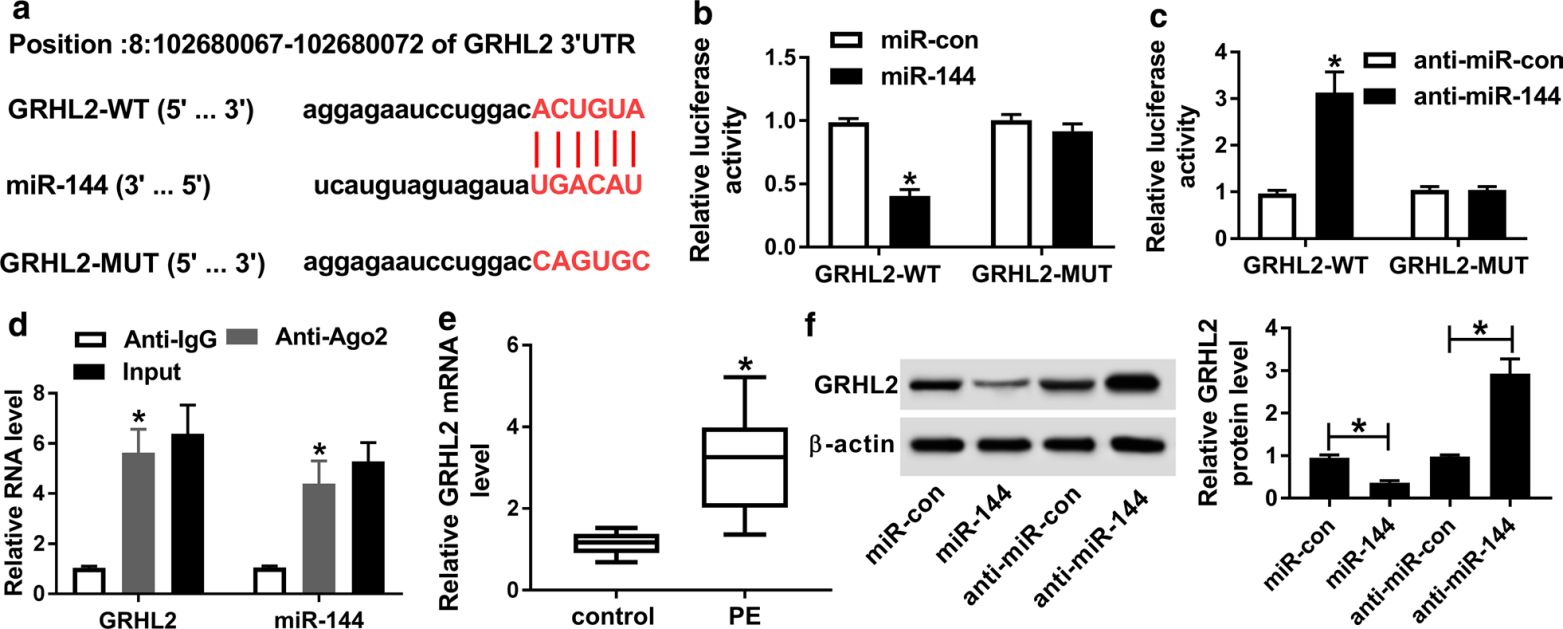
GRHL2 was a direct target of miR-144. **a** Schematic of the complementary site for miR-144 in the 3′-UTR of GRHL2 and the mutation of the seed region. **b**, **c** Relative luciferase activity in HTR-8/SVneo cells introduced with GRHL2-WT or GRHL2-MUT together with miR-con mimic, miR-144 mimic, anti-miR-con or anti-miR-144. **d** The enrichment of GRHL2 and miR-144 in the RISC of HTR-8/SVneo cells using anti-Ago2 or IgG antibody, with Input content as positive control. **e** qRT-PCR for GRHL2 mRNA in placental tissues from 26 PE patients and 15 healthy volunteers. **f** Western blot for GRHL2 protein level in HTR-8/SVneo cells transfected with miR-con mimic, miR-144 mimic, anti-miR-con, or anti-miR-144. *P < 0.05

Next, using microT-CDS online software, a predicted miR-144-binding site was identified in the 3′-UTR of GRHL2 (Fig. 4a). To validate whether GRHL2 was a direct target of miR-144, we carried out dual-luciferase reporter and RIP assays. As shown in Fig. 4b, c, the luciferase activity of GRHL2 3′-UTR reporter construct was significantly down-regulated by miR-144 overexpression and up-regulated following miR-144 silencing. However, these effects were highly abrogated by the mutation of the seed region (Fig. 4b, c). Moreover, anti-Ago2 antibody synchronously induced the significant enhancement in the enrichment of GRHL2 and miR-144 (Fig. 4d). The data of qRT-PCR assay also demonstrated a strong up-regulation of GRHL2 mRNA level in PE placental tissues compared with the healthy group (Fig. 4e). Moreover, GRHL2 protein level was strikingly reduced by a high miR-144 expression, while it was prominently elevated after miR-144 silencing in HTR-8/SVneo cells (Fig. 4f).

### The regulatory effects of miR-144 on trophoblast cell proliferation, migration and invasion were reversed by GRHL2 expression alteration

**Fig. 5:**
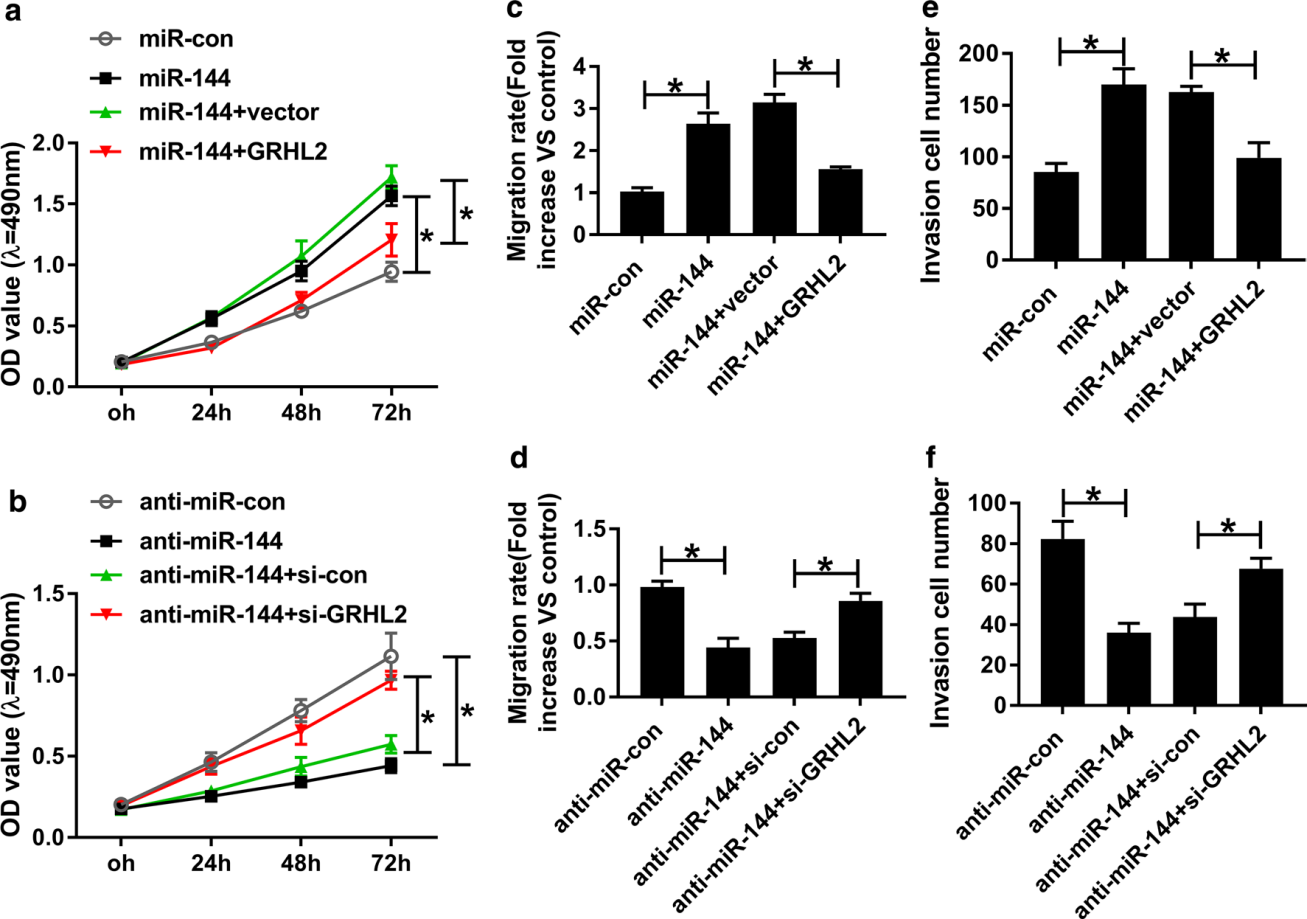
GRHL2 mediated the regulatory effects of miR-144 on trophoblast cell proliferation, migration and invasion. Cell proliferation by MTS assay (**a**, **b**), cell migration by wound healing assay (**c**, **d**), cell invasion by transwell assay (**e**, **f**) in HTR-8/SVneo cells transfected with miR-con mimic, miR-144 mimic, miR-144 mimic + negative control plasmid (vector), miR-144 mimic + GRHL2 overexpression plasmid, anti-miR-con, anti-miR-144, anti-miR-144 + si-con, or anti-miR-144 + si-GRHL2. *P < 0.05

To provide further mechanistic insight into the link between miR-144 and GRHL2 in PE pathogenesis, HTR-8/SVneo cells were transfected with miR-144 mimic + GRHL2 overexpression plasmid or anti-miR-144 + si-GRHL2. In comparison to the negative group, cell proliferation was highly facilitated following miR-144 overexpression (Fig. 5a) and hindered by a low expression of miR-144 (Fig. 5b). Furthermore, miR-144 overexpression resulted in the enhancement in cell migration and invasion, while miR-144 depletion showed opposite effects (Fig. 5c–f). More interestingly, the regulatory effects of miR-144 overexpression and depletion were strongly abolished by the contransfection of GRHL2 overexpression plasmid or si-GRHL2 (Fig. 5a–f).

### CircZDHHC20 protected against GRHL2 repression through sequestering miR-144

**Fig. 6:**
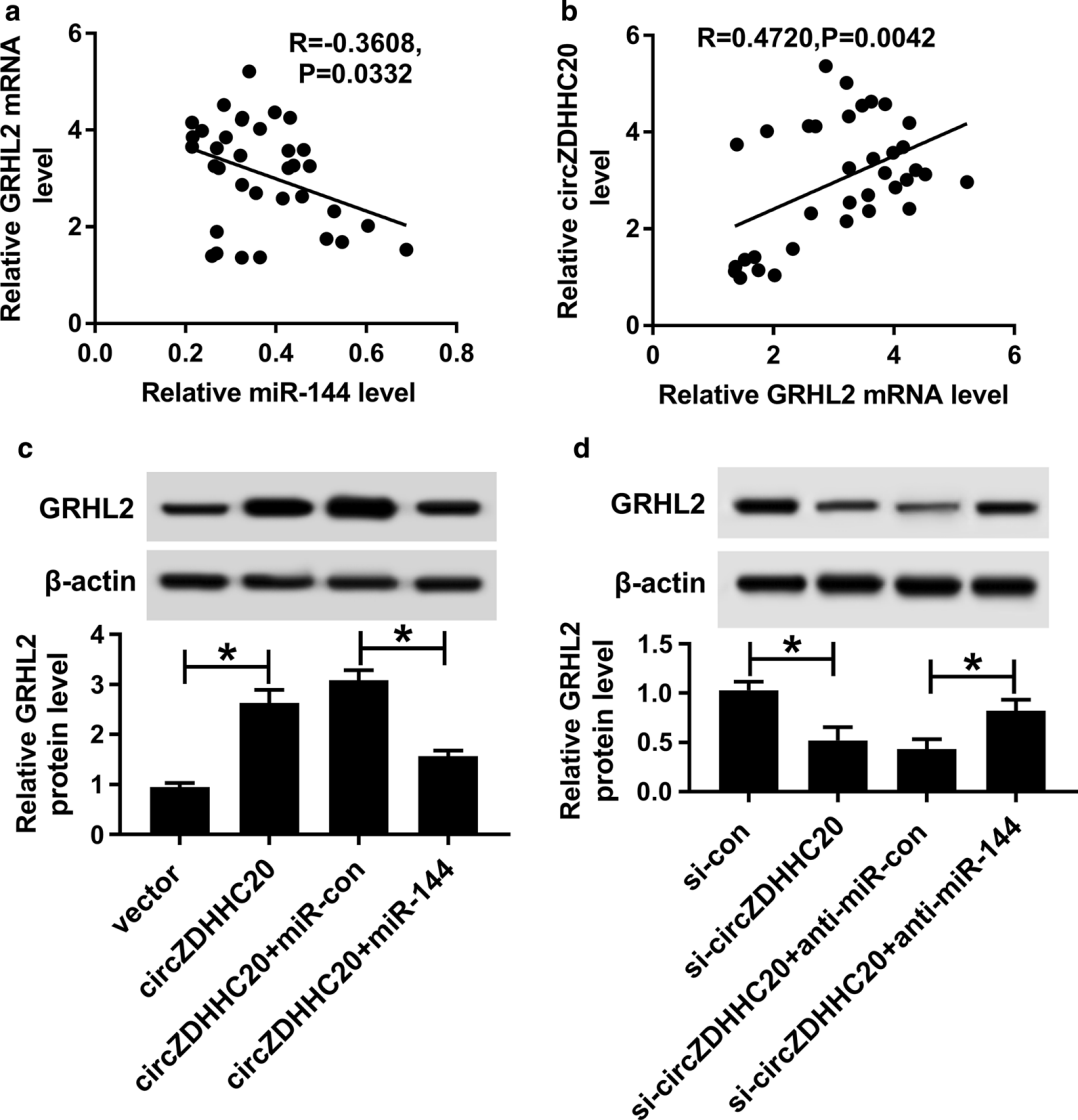
CircZDHHC20 regulated GRHL2 expression through sponging miR-144. Correlations between GRHL2 mRNA expression and miR-144 level (**a**) or circZDHHC20 level (**b**) in 26 placental tissues from PE patients using the Spearman test. **c**, **d** GRHL2 protein level by western blot in HTR-8/SVneo cells transfected with negative control plasmid (vector), circZDHHC20 overexpression plasmid (circZDHHC20), circZDHHC20 + miR-con mimic, circZDHHC20 + miR-144 mimic, si-con, si-circZDHHC20, si-circZDHHC20 + anti-miR-con or si-circZDHHC20 + anti-miR-144. *P < 0.05

Lastly, we investigated whether, if so, how circZDHHC20 modulated GRHL2 expression in HTR-8/SVneo cells. qRT-PCR results revealed that GRHL2 expression was inversely correlated with miR-144 level and positively correlated with circZDHHC20 expression in placental tissues from PE patients (Fig. 6a, b). As expected, in comparison to their counterparts, GRHL2 protein expression was significantly increased by highly expressed circZDHHC20 (Fig. 6c), while it was remarkably decreased when circZDHHC20 deficiency (Fig. 6d). Nevertheless, these effects were strongly reversed in response to miR-144 level alteration (Fig. 6c, d).

## Discussion

It has become increasingly clear that many circRNAs are abnormally expressed in PE placenta, providing a novel avenue of research regarding the pathogenesis of PE [10, 20]. However, the potential function and underlying mechanisms of these circRNAs in PE development are not yet well understood. The database of online software algorithms predicted a possible ceRNA network of the circZDHHC20/miR-144/GRHL2 axis in PE. The present work had led to the identification of circZDHHC20 that suppressed the proliferation, migration, and invasion in trophoblast cells through sponging miR-144 and up-regulating GRHL2.

In the current project, our data firstly validated a significant up-regulation of circZDHHC20, consistent with previous work [10]. To verify that circZDHHC20 was indeed circular transcript, we carried out RNase R assay and reverse transcription experiments with Oligo(dT)_18_ primers on account of the inherent stability by their exonuclease resistance and the depletion of the 3′ pA tail, respectively. As expected, our data indicated that circZDHHC20 was resistant to RNase R digestion, and its level was lower when Oligo(dT)_18_ primers were used. Since circRNAs prominently located in the cytoplasm could interact with available miRNAs [16, 18], we then validated the subcellular localization of circZDHHC20 in HTR-8/SVneo cells, and our data demonstrated that circZDHHC20 was predominantly present in the cytoplasm. To investigate the role of circZDHHC20 in PE pathogenesis, loss-of-function and gain-of-function experiments were performed. We were first to uncover that circZDHHC20 overexpression significantly retarded trophoblast cell proliferation, migration, and invasion, while its deficiency exhibited opposite effects. Increasing evidence has demonstrated that an increase in trophoblast migration/invasion restraining factors contributes to PE aggressiveness [21]. Based on the above, highly expressed circZDHHC20 was associated with PE pathogenesis.

Of particular interest, circRNAs regulate the abundance of specific miRNAs through mechanisms including sequestration. To confirm whether circZDHHC20 could act as miRNAs sponges, the CircInteractome computational method was used. Among these candidates, miR-144 was selected for further exploration owing to its significant down-regulation in PE placenta and maternal plasma [13–15]. As for miR-144, it was reported to regulate tumor cell proliferation, migration and invasion in a series of human cancers, such as breast cancer, pancreatic cancer, and nasopharyngeal carcinoma [22–24]. In the present work, we firstly validated that circZDHHC20 sequestered miR-144 through acting as a miR-144 sponge. Our data also indicated that miR-144 level was reduced and inversely correlated with circZDHHC20 expression in PE placental tissues. Moreover, miR-144 overexpression enhanced trophoblast cell proliferation, migration, and invasion, and its silencing showed opposite effects, in line with an earlier study [15]. More importantly, our results substantiated that miR-144 mediated the regulation of circZDHHC20 on proliferation, migration, and invasion in trophoblast cells.

Then, we used online software microT-CDS to help identify the molecular targets of miR-144. Interestingly, GRHL2 harbored a putative complementary sequence for miR-144. GRHL2 has been established as a tumor server through the regulation of cell proliferation, migration, and invasion in multiple cancers, including ovarian cancer and gastric cancer [25, 26]. Moreover, GRHL2 hampered the differentiation of keratinocyte through reducing related protein expression via epigenetic mechanism [27]. In the present research, we firstly uncovered that GRHL2 was a direct target of miR-144 in trophoblast cells. Our data by qRT-PCR also demonstrated a significant up-regulation of GRHL2 level in PE placenta. Moreover, we validated that miR-144 regulated trophoblast cell proliferation, migration, and invasion by targeting GRHL2. Additionally, our data underscored that circZDHHC20 regulated GRHL2 repression through acting as a sponge of miR-144. Similar to our findings, Shen et al. reported that circTRNC18 accelerated PE progression via sponging miR-762 and modulating GRHL2 expression [9].

## Conclusion

In conclusion, our study suggested that circZDHHC20 inhibited the proliferation, migration, and invasion in trophoblast cells partially through targeting miR-144/GRHL2 axis. Our present work provided a novel mechanism involved in PE pathogenesis.

## Data Availability

All data generated or analyzed during this study are included in this published article.
